# Deficiency of NOD1 Improves the β-Adrenergic Modulation of Ca^2+^ Handling in a Mouse Model of Heart Failure

**DOI:** 10.3389/fphys.2018.00702

**Published:** 2018-06-14

**Authors:** Almudena Val-Blasco, Jose A. Navarro-García, Maria Tamayo, Maria J. Piedras, Patricia Prieto, Carmen Delgado, Gema Ruiz-Hurtado, Laura Rozas-Romero, Marta Gil-Fernández, Carlos Zaragoza, Lisardo Boscá, María Fernández-Velasco

**Affiliations:** ^1^Innate Immune Response Group, Instituto de Investigación Hospital Universitario La Paz, La Paz University Hospital, Madrid, Spain; ^2^Cardiorenal Translational Laboratory and Hypertension Unit, Institute of Research i+12, Hospital Universitario 12 de Octubre, Madrid, Spain; ^3^Departamento de Bioquímica, Facultad de Medicina, Instituto de Investigaciones Biomédicas Alberto Sols, Consejo Superior de Investigaciones Científicas, Madrid, Spain; ^4^Department of Anatomy, Faculty of Health Sciences, Francisco de Vitoria University (UFV), Pozuelo de Alarcón, Spain; ^5^Unidad de Investigación Cardiovascular, Universidad Francisco de Vitoria, Hospital Universitario Ramón y Cajal (IRYCIS), CIBERCV, Madrid, Spain

**Keywords:** β-adrenergic response, heart failure, NOD1, Ca^2+^ handling, innate immune system

## Abstract

Heart failure (HF) is a complex syndrome characterized by cardiac dysfunction, Ca^2+^ mishandling, and chronic activation of the innate immune system. Reduced cardiac output in HF leads to compensatory mechanisms via activation of the adrenergic nervous system. In turn, chronic adrenergic overstimulation induces pro-arrhythmic events, increasing the rate of sudden death in failing patients. Nucleotide-binding oligomerization domain-containing protein 1 (NOD1) is an innate immune modulator that plays a key role in HF progression. NOD1 deficiency in mice prevents Ca^2+^ mishandling in HF under basal conditions, but its role during β-adrenergic stimulation remains unknown. Here, we evaluated whether NOD1 regulates the β-adrenergic modulation of Ca^2+^ signaling in HF. Ca^2+^ dynamics were examined before and after isoproterenol perfusion in cardiomyocytes isolated from healthy and from post-myocardial infarction (PMI) wild-type (WT) and *Nod1*^-/-^ mice. Isoproterenol administration induced similar effects on intracellular [Ca^2+^]_i_ transients, cell contraction, and sarcoplasmic reticulum (SR)-Ca^2+^ load in healthy WT and *Nod1*^-/-^ cells. However, compared with WT-PMI cells, isoproterenol exposure induced a significant increase in the [Ca^2+^]_i_ transients and cell contraction parameters in *Nod1*^-/-^-PMI cells, which mainly due to an increase in SR-Ca^2+^ load. NOD1 deficiency also prevented the increase in diastolic Ca^2+^ leak (Ca^2+^ waves) induced by isoproterenol in PMI cells. mRNA levels of β1 and β2 adrenergic receptors were significantly higher in *Nod1*^-/-^-PMI hearts vs WT-PMI hearts. Healthy cardiomyocytes pre-treated with the selective agonist of NOD1, iE-DAP, and perfused with isoproterenol showed diminished [Ca^2+^]_i_ transients amplitude, cell contraction, and SR-Ca^2+^ load compared with vehicle-treated cells. iE-DAP-treated cells also presented increased diastolic Ca^2+^ leak under β-adrenergic stimulation. The selectivity of iE-DAP on Ca^2+^ handling was validated by pre-treatment with the inactive analog of NOD1, iE-Lys. Overall, our data establish that NOD1 deficiency improves the β-adrenergic modulation of Ca^2+^ handling in failing hearts.

## Introduction

Heart failure (HF) is a complex clinical disorder characterized by the inability of the heart to deliver blood and nutrients to metabolic tissues. Chronic HF is a progressive disease, with high morbidity and mortality, and poses a significant economic burden on the healthcare system. Given its poor prognosis, the identification of molecular pathways contributing to HF is a major research goal.

Compensatory activation of the adrenergic nervous system is a pathophysiological response to HF progression, which functions to maintain cardiac homeostasis through activation of neural hormones, mainly catecholamines (Gordan et al., 2015). Accordingly, sympathetic hyperactivity is a hallmark of HF, activating β-adrenergic receptors to increase heart rate and cardiac contractility via excitation–contraction (EC) coupling, in an attempt to counteract the decreased cardiac output. Chronic exposure of the heart to elevated levels of catecholamines may, however, ultimately lead to the desensitization of β-adrenergic receptors, exacerbating the loss of cardiac function and increasing the risk for triggered arrhythmias and sudden death (Feldman et al., 2005; Brum et al., 2006; Venetucci et al., 2008).

There is a growing body of evidence suggesting a link between the innate immune response and HF progression (Lin and Knowlton, 2014; Mann, 2015). Whether the myocardial inflammatory response impairs cardiac damage is a question of broad significance. Recent studies indicate that some receptors of the innate immune system, including nucleotide-binding oligomerization domain-like receptors (NLRs), play significant roles in the host response after cardiac damage (Bullón et al., 2016; Monnerat et al., 2016; Val-Blasco et al., 2017a). Nucleotide-binding oligomerization domain (NOD) receptors are NLR family members that recognize conserved motifs of bacterial peptidoglycans in many Gram-negative bacteria (Caruso et al., 2014). Beyond their role as microbial pattern recognition receptors, it has recently been described that NODs can also be activated by non-infectious factors such as endoplasmic reticulum stress (Keestra-Gounder et al., 2016). Ligand activation of NOD receptors induces a conformational change in the protein, leading to self-oligomerization and promoting the recruitment of its adaptor, receptor-interacting protein 2 (RIP2), which activates nuclear factor-κB and triggers the inflammatory response (Park et al., 2007). NOD-containing protein 1 (NOD1) is expressed in the heart, and is functional in resident fibroblasts and cardiomyocytes (Fernández-Velasco et al., 2012; Delgado et al., 2015). Several studies have demonstrated a role for NOD1 in the progression of cardiovascular diseases, including atherosclerosis and diabetic cardiomyopathy (Prieto et al., 2014; Kanno et al., 2015; Val-Blasco et al., 2017b). We have recently shown that NOD1 is upregulated in both mouse and human failing myocardium, and its genetic deletion or pharmacological blockade prevents cardiac dysfunction and deleterious remodeling in failing hearts principally by preventing the HF-related Ca^2+^ mishandling (Val-Blasco et al., 2017a). Conversely, activation of NOD1 promotes a dysregulation of the intracellular Ca^2+^ dynamics, similar to those observed in HF (Delgado et al., 2015).

Ca^2+^ plays a key role in cardiac EC coupling. During EC coupling, an action potential leads to a small increase in intracellular Ca^2+^ via activated sarcolemmal L-type Ca channels (I_CaL_), and this is amplified by a greater release of Ca^2+^ from the sarcoplasmic reticulum (SR) by ryanodine receptors (RyR_2_), increasing the intracellular Ca^2+^ concentration ([Ca^2+^]_i_) – a process known as Ca^2+^-induced Ca^2+^ release. This elevation in [Ca^2+^]_i_ results in myofilament activation and cell contraction. During relaxation, Ca^2+^ is removed from the cytosol primarily by two mechanisms: Ca^2+^ re-uptake by the SR-ATPase (SERCA2a) and Na^+^/Ca^2+^ exchanger activation.

Excitation–contraction coupling is modulated by the sympathetic nervous system through activation of the β-adrenergic receptor coupled to Gs-type G-proteins, promoting an elevation in the intracellular concentration of cAMP (Bers, 2002). In turn, cAMP activates protein kinase A (PKA), resulting in the phosphorylation of several EC coupling-Ca^2+^ transporters such as I_CaL_, RyR_2_, and phospholamban, among others (Mayourian et al., 2018).

Several lines of evidence have established a direct relationship between Ca^2+^ dysregulation and HF progression (Gómez et al., 1997; Heinzel et al., 2011; Ruiz-Hurtado et al., 2015). Depressed systolic Ca^2+^ release, the decline in the SR-Ca^2+^ load, and the increased Ca^2+^ diastolic leak seem to be key players in HF progression, and these changes worsen under chronic β-adrenergic activation. We recently showed that NOD1 modulates intracellular Ca^2+^ handling in human and experimental failing hearts (Val-Blasco et al., 2017a); however, the role of NOD1 in the modulation of Ca^2+^ handling under β-adrenergic stimulation has not been investigated. Using a mouse HF model, we determined whether the deficiency of NOD1 impairs the modulation of Ca^2+^ dynamics under β-adrenergic stimulation.

## Materials and Methods

### Myocardial Infarction Model

Male *Nod1*^-/-^ mice on a C57BL/6J (6B; 129P2-*Nod1*^tm1Nnz^/J) background were used in this study (Chamaillard et al., 2003). As controls, we used wild-type (WT) C57BL/6J mice (The Jackson Laboratory, Bar Harbor, ME, United States). Experiments involving mice were carried out in compliance with Spanish and European guidelines (2010/63/EU) regarding animal policy and welfare recommendations. The present study was performed in male mice because they have been reported to be more prone than female to develop cardiac injury and Ca^2+^ mishandling after isoproterenol administration in experimental models of HF (Cross et al., 2002).

Mice were anesthetized by intraperitoneal (i.p.) injection of a mixture of ketamine (Imalgene^®^, 70 mg/kg) and xylazine (Rompun^®^, 10 mg/kg). Unconscious mice were shaved in the anterior region of the neck and the chest, intubated by tracheostomy, and connected to a small animal ventilator (MiniVent type 845, Harvard Apparatus) for artificial ventilation at 150 strokes/minute and 230 μL stroke volume. A 2–3 mm incision parallel to the lower costal edge was made and the left pectoralis major muscle was dissociated until the ribs were exposed. Left thoracotomy was performed between the third and fourth ribs to visualize the anterior surface of the heart and left lung. A 1-mm-thick piece of gelatin sponge (Spongostan^®^), slightly moistened with saline, was inserted through the hollow thoracotomy to protect the lung. The ribs were separated with an eyelid-retractor and a branch of the left coronary artery was ligated with a blue polypropylene monofilament surgical non-absorbable suture 6/0. The Spongostan^®^ sponge was then removed and the chest was closed with braided silk non-absorbable suture 4/0. Respiratory stimulation was performed to return the heart to spontaneous breathing. For postoperative analgesia, buprenorphine (Buprex^®^, 0.05 mg/kg) was applied subcutaneously. Mice were kept on a warm electric blanket until spontaneous recovery. Sham-operated mice were used as the control group and underwent the same procedure, but without coronary ligation.

### Myocyte Isolation

Adult male mice (2 months of age) were anesthetized with sodium pentobarbital (100 mg/kg i.p.) and heparinized (4 UI/g i.p.). Hearts were rapidly removed and cannulated via the ascending aorta for Langendorff perfusion. Retrograde perfusion was initiated with a free calcium Tyrode solution containing 0.2 mM EGTA over 2–3 min at room temperature, and subsequently with Tyrode solution containing CaCl_2_ (0.1 mM) and type II collagenase (1 mg/mL; Worthington Biochemical, Lakewood, NY, United States). Successful digestion was assumed when the flux rate increased and the heart color changed from red to pallor. The heart was then taken off the apparatus, and the ventricles were removed, minced into small pieces, and mechanically dissociated in a thermostatic bath at 37°C in the enzymatic solution. The cardiomyocyte cell suspension was filtered through a nylon mesh (250 μm) and centrifuged at 300 rpm for 3 min. The cell pellet was suspended in Tyrode solution containing CaCl_2_ (0.5 mM) and centrifuged as before. Finally, the cells were suspended in Tyrode solution containing CaCl_2_ (1 mM). The Tyrode solution contained (in mM): 130 NaCl, 5.4 KCl, 0.5 MgCl_2_, 25 HEPES, 0.4 NaH_2_PO_4_, and 22 glucose, which was adjusted to pH 7.4 with NaOH.

### Confocal Microscopy

Local increases in intracellular Ca^2+^ concentration, [Ca^2+^]_i_ transients, and Ca^2+^ waves were analyzed in intact isolated cardiomyocytes loaded with the fluorescent Ca^2+^ dye Fluo-3AM (5 μmol/L, Invitrogen). [Ca^2+^]_i_ transients were recorded in cells electrically excited at 2 Hz by field stimulation using two parallel platinum electrodes. Ca^2+^ waves were acquired in quiescent myocytes. SR Ca^2+^ load was assessed by rapid caffeine application (10 mM) in cells previously excited at 2 Hz.

Images were acquired by confocal microscopy (Meta Zeiss LSM 710, 40× oil immersion objective with a 1.2 NA), by scanning the cell with an argon laser every 1.54 ms. Fluo-3AM was excited at 488 nm and emitted fluorescence was collected at >505 nm. Data analysis was performed with homemade routines using software written on the IDL platform (Research System Inc., Boulder, CO, United States), designed by Dr. AM Gómez (Inserm, UMR-S 1180). Images were corrected for background fluorescence. The fluorescence values (*F*) were normalized by the basal fluorescence (*F*0) to obtain the fluorescence ratio (*F*/*F*0).

### Drugs and Treatments

Isolated cardiomyocytes were perfused with 10^-8^ M isoproterenol (Sigma-Aldrich, Madrid, Spain) for 1–5 min and intracellular Ca^2+^ dynamics were recorded. In some experiments, cells were pretreated with the selective NOD1 agonist C12-iE-DAP (iE-DAP, InvivoGen, San Diego, CA, United States), which has significant cell membrane permeability and high potency (Fernández-Velasco et al., 2012; Delgado et al., 2015; Val-Blasco et al., 2017a), or iE-Lys (Invivogen), an inactive analog of NOD1. Nodinitib-1 (Cayman) was used as a selective NOD1 inhibitor and mice were injected IP with 5 μmol/L nodinitib-1 or vehicle (<0.01% DMSO) three times weekly for 6 weeks.

### Real-Time PCR

Frozen heart tissue was pulverized to powder and then homogenized in TRIZOL Reagent^®^ solution (Ambion) with a Polytron system (MWR). RNA (250 ng) was reverse transcribed to cDNA using the *High-Capacity cDNA Reverse Transcription Kit* (Applied Biosystems #4368813). This template cDNA was used in the qPCR reaction with *Power SYBR Green PCR Master* mix (Applied Biosystems #4367659) and specific primers in a 7900HT Fast Real Time PCR system (Applied Biosystems). 18s-RNA was used as a housekeeping gene. The primer sequences (5′-3′) for β1 and β2 adrenergic receptors and 18s-RNA were as follows:

β1-F CGCTGATCTGGTCATGGGAT
β1-R GAAGAAGGAGCCGTACTCCC
β2-F AATAGCAACGGCAGAACGGA
β2-R TCACAAAGCCTTCCATGCCT
18s-F CCAGTAAGTGCGGGTCATAAGC
18s-R CCTCACTAAACCATCCAATCGG

### Statistical Analysis

Results are reported as mean ± SEM. Statistical analysis was performed using two-way analysis of variance, paired (two-sided) Student’s t test or χ^2^ test, as appropriate. All statistical analyses were performed with Origin 8.0 software (OriginLab, Northampton, MA, United States). Significance was set at *P* < 0.05.

## Results

### Hypertrophy Development in Mice Subjected to Myocardial Infarction

Compared with healthy WT mice, post-myocardial infarction (PMI)-mice 6 weeks after surgery showed significantly increased heart weight, heart weight/body weight ratio, and heart weight/tibia length ratio, consistent with the development of cardiac hypertrophy (**Table 1**). By contrast, *Nod1*^-/-^-PMI mice showed similar heart weight, heart weight/body weight, and heart weight/tibia length values to *Nod1*^-/-^ mice (**Table 1**). These data indicate that deficiency of NOD1 attenuates cardiac hypertrophy in our PMI mouse model.

**Table 1:** Cardiac properties of wild-type and *Nod1-deficent* mice.

	WT (*N* = 5)	*Nod1*^-/-^ (*N* = 5)	WT-PMI (*N* = 4)	*Nod1*^-/-^-PMI (*N* = 4)
HW (mg)	187.20 ± 3.72	173.60 ± 4.79	272.00 ± 10.70^∗∗∗^	193.5 ± 13.35^&&^
BW (g)	29.71 ± 1.37	29.95 ± 1.14	31.47 ± 0.54	30.03 ± 1.14
TL (mm)	21.90 ± 0.40	21.50 ± 0.31	21.25 ± 0.47	21.50 ± 0.28
HW/TL (mg/mm)	8.56 ± 0.25	8.08 ± 0.27	12.81 ± 0.53^∗∗∗^	9.01 ± 0.66^&&^
HW/BW (mg/g)	6.35 ± 0.32	5.82 ± 0.22	8.65 ± 0.37^∗∗^	6.36 ± 0.26^&&^

*HW, heart weight; BW, body weight; TL, tibia length. ^∗∗^*P* < 0.01, ^∗∗∗^*P* < 0.001 vs WT and ^&&^*P* < 0.01 vs WT-PMI.*

### Deficiency of NOD1 Improves the β-Adrenergic Modulation of Systolic Ca^2+^ Release and Sarcoplasmic Reticulum Ca^2+^ Load in PMI Cardiomyocytes

**FIGURE 1:**
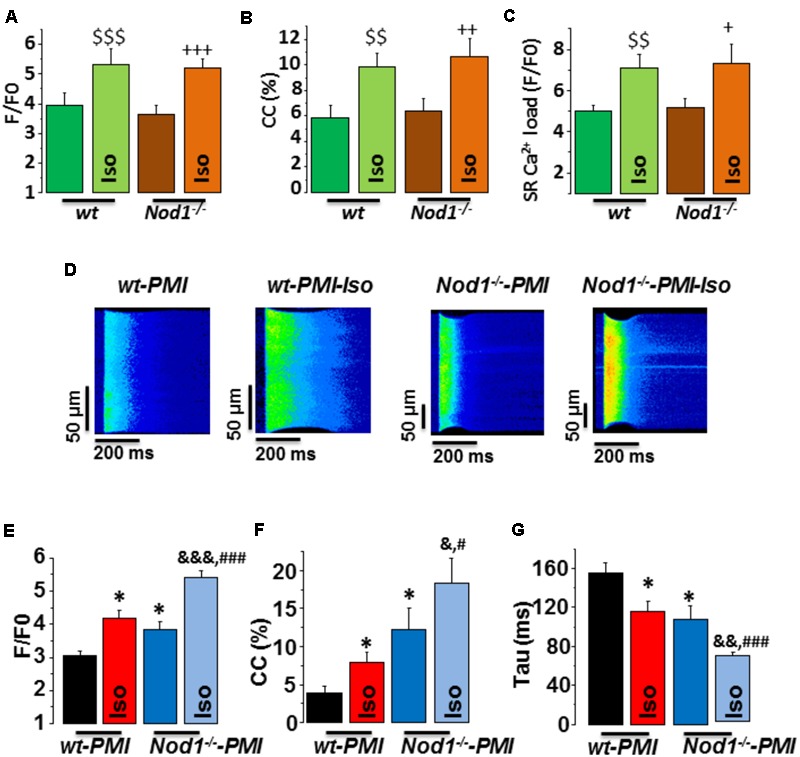
Deficiency of NOD1 improves the modulation of systolic Ca^2+^ handling and SR-Ca^2+^ load by β-adrenergic stimulation in cardiomyocytes from failing mice. **(A–C)** Mean values of amplitude of [Ca^2+^]_i_ transients (*F*/*F*0, **A**), cell contractions (CC, %, **B**), and caffeine-evoked [Ca^2+^]_i_ transients amplitude (SR-Ca^2+^ load, *F*/*F*0, **C**) in WT (*n* = 13 cells/5 mice) and *NOD1*^-/-^ cardiomyocytes (*n* = 14 cells/4 mice) with and without 10^-8^ M isoproterenol (Iso). **(D)** Representative line-scan confocal images of [Ca^2+^]_i_ transients obtained from WT-PMI and *NOD1*^-/-^-PMI cardiomyocytes with and without Iso perfusion. **(E,F)** Mean values of peak fluorescence [Ca^2+^]_i_ transients (*F*/*F*0, **E**), cell contraction (CC, %, **F**), and decay time of [Ca^2+^]_i_ transients (tau, ms, **G**) in WT-PMI (*n* = 12 cells/4 mice) and *NOD1*^-/-^-PMI (*n* = 13 cells/4 mice) cardiomyocytes with and without Iso. Histograms represent the mean ± SEM: ^$$^*P* < 0.01, ^$$$^*P* < 0.001 vs WT; ^+^*P* < 0.05 vs *NOD1*^-/-^, ^++^*P* < 0.01 vs *NOD1*^-/-^, ^+++^*P* < 0.001 vs *NOD1*^-/-^; ^∗^*P* < 0.05 vs WT-PMI; ^&^*P* < 0.05, ^&&^*P* < 0.01, ^&&&^*P* < 0.001 vs *NOD1*^-/-^-PMI; and ^#^*P* < 0.05, ^###^*P* < 0.001 vs WT-PMI treated with Iso.

We first analyzed whether deficiency of NOD1 determined the β-adrenergic response to Ca^2+^ handling in healthy myocytes. Isoproterenol perfusion induced a similar increase in [Ca^2+^]_i_ transients amplitude (**Figure 1A**), cell contraction (**Figure 1B**), and SR-Ca^2+^ load (**Figure 1C**) in WT and *Nod1*^-/-^ cardiomyocytes.

Given that β-adrenergic regulation of Ca^2+^ dynamics plays a key role in HF-related cardiac dysfunction, we next questioned whether NOD1 deficiency impacted isoproterenol-induced modulation of Ca^2+^ handling in cardiomyocytes after PMI. **Figure 1D** shows representative line-scan Ca^2+^ fluorescence images obtained after electric field stimulation at 2 Hz in all groups. Under β-adrenergic stimulation, both WT-PMI and *Nod1*^-/-^-PMI cardiomyocytes exhibited increased [Ca^2+^]_i_ transients (**Figure 1E**), augmented cell contraction (**Figure 1F**), and faster time decay of [Ca^2+^]_i_ transients (**Figure 1G**), but all these changes were significantly greater in *Nod1*^-/-^-PMI cells. These results reveal that, compared with WT-PMI cells, *Nod1*^-/-^-PMI myocytes show a significant improvement in Ca^2+^ mishandling, which was mostly due to the increased amplitude of [Ca^2+^]_i_ transients (**Figures 1D,E**) and also higher cell contraction parameters (**Figure 1F**). In this line, mean values of the amplitude of [Ca^2+^]_i_ transients (*F*/*F*0) in cardiomyctes obtained under isoproterenol administration were 5.31 ± 0.52 (*n*/*N* = 13/5) in WT, 5.18 ± 0.32 (*n*/*N* = 14/4) in *Nod1*^-/-^, 4.18 ± 0.22 (*n*/*N* = 12/4) in WT-PMI, and 5.41 ± 0.21 (*n*/*N* = 13/4) in *Nod1*^-/-^-PMI (**Figures 1A,E**). Thus, [Ca^2+^]_i_ transients obtained in the *Nod1*^-/-^-PMI group during isoproterenol perfusion were similar to those of equivalent healthy WT and *Nod1*^-/-^ cardiomyocytes. Consistent with this finding, treatment of WT-PMI mice with the selective NOD1 inhibitor nodinitib-1 resulted in higher amplitude isoproterenol-induced [Ca^2+^]_i_ transients in isolated cardiomyocytes compared with cells isolated from vehicle-treated mice (Supplementary Figure S1).

**FIGURE 2:**
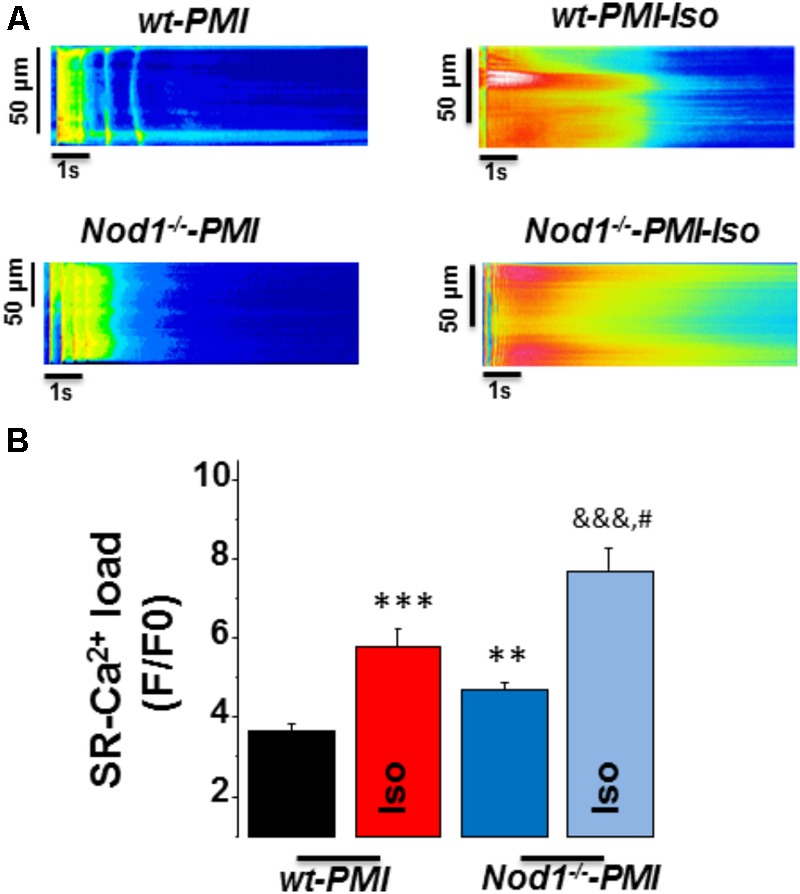
Deficiency of NOD1 increases the β-adrenergic response on SR-Ca^2+^ load in cardiomyocytes from failing mice. **(A)** Representative line-scan confocal images of caffeine-evoked [Ca^2+^]_i_ transients obtained from WT-PMI and *NOD1*^-/-^-PMI cardiomyocytes with and without Iso perfusion. **(B)** Mean values of caffeine-evoked [Ca^2+^]_i_ transients amplitude (SR-Ca^2+^ load, *F*/*F*0) in WT-PMI (*n* = 23 cells/4 mice), *NOD1*^-/-^-PMI (*n* = 19 cells/4 mice), WT-PMI (*n* = 13 cells/4 mice), and *NOD1*^-/-^-PMI (*n* = 12 cells/4 mice) cardiomyocytes treated with Iso. Histograms represent the mean ± SEM: ^∗∗^*P* < 0.01, ^∗∗∗^*P* < 0.001 vs WT-PMI; ^&&&^*P* < 0.001 vs *NOD1*^-/-^-PMI; and ^#^*P* < 0.05 vs WT-PMI cardiomyocytes treated with Iso.

Next, to question whether changes in [Ca^2+^]_i_ transients during β-adrenergic stimulation in PMI cardiomyocytes were related to modifications in SR-Ca^2+^ load, we measured caffeine-evoked [Ca^2+^]_i_ transients in all groups. **Figure 2A** shows examples of line-scan images of caffeine-evoked [Ca^2+^]_i_ transients obtained in each group. Mean values of the amplitude of caffeine-evoked [Ca^2+^]_i_ transients (F/F0) in cardiomyocytes during isoproterenol exposure was 7.07 ± 0.69 (*n*/*N* = 9/3) in WT, 7.34 ± 0.93 (*n*/*N* = 10/4) in *Nod1*^-/-^ (**Figure 1C**), 5.77 ± 0.46 (*n*/*N* = 13/4) in WT-PMI, and 7.40 ± 0.50 (*n*/*N* = 12/4) in *Nod1*^-/-^-PMI (**Figure 2B**). Thus, the amplitude of [Ca^2+^]_i_ transients triggered by caffeine was significantly higher in *Nod1*^-/-^-PMI mice than in WT-PMI mice, both in the absence or the presence of isoproterenol (**Figure 2B**). Additionally, the high values of SR-Ca^2+^ load in the *Nod1*^-/-^-PMI group treated with isoproterenol were similar to those found in healthy WT and *Nod1*^-/-^ cells treated with isoproterenol.

Taken together, β-adrenergic stimulation induced better systolic Ca^2+^ release and cell contractility in *Nod1*^-/-^-PMI cells than in WT-PMI cells. These effects correlated with the significant increase in the SR-Ca^2+^ load induced by isoproterenol in *Nod1*^-/-^-PMI cells.

### Deficiency of NOD1 Prevents the Increased Diastolic Ca^2+^ Release Induced by β-Adrenergic Stimulation in PMI Cardiomyocytes

Since SR-Ca^2+^ load impairment is closely related to changes in diastolic Ca^2+^ release, we analyzed spontaneous Ca^2+^ waves in quiescent cells.

**FIGURE 3:**
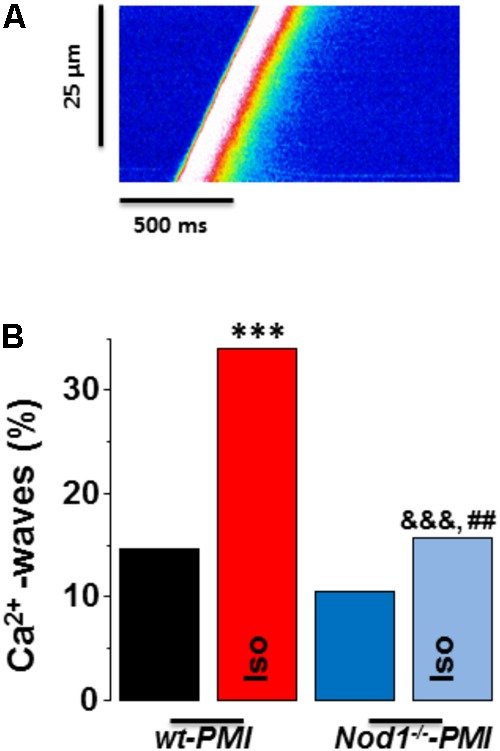
Deficiency of NOD1 prevents the increase in diastolic Ca^2+^ release induced by isoproterenol in cardiomyocytes from failing mice. **(A)** A representative line-scan image of a Ca^2+^ wave recording from a WT-PMI myocyte. **(B)** The mean data for Ca^2+^ wave occurrence from WT-PMI (*n* = 9 cells/3 mice) and NOD1^-/-^-PMI (*n* = 7 cells/3 mice) cardiomyocytes with and without Iso. Histograms represent the mean ± SEM: ^∗^*P* < 0.05, ^∗∗∗^*P* < 0.001, vs WT-PMI; ^&^*P* < 0.05, ^&&&^*P* < 0.001 vs *NOD1*^-/-^-PMI; and ^##^*P* < 0.05 vs WT-PMI cardiomyocytes treated with Iso.

**Figure 3A** shows a representative line-scan image of a Ca^2+^ wave recording from a WT-PMI myocyte. Ca^2+^ wave occurrence analysis (**Figure 3B**) revealed no statistically significant changes between WT-PMI (14.60%) and *Nod1*^-/-^-PMI (10.50%) cells under basal conditions. By contrast, both groups showed a significant elevation in the Ca^2+^ wave rate after isoproterenol perfusion, but this was significantly higher in WT-PMI cardiomyocytes perfused with isoproterenol (**Figure 3**). Accordingly, the percentage of cells with Ca^2+^ waves during isoproterenol exposure was 34.07% in WT-PMI cells and 15.65% in *Nod1*^-/-^-PMI cells (*P* < 0.01).

These results indicate that deficiency of NOD1 prevents the increase of Ca^2+^ diastolic leak induced by β-adrenergic stimulation in PMI cardiomyocytes, thus providing an explanation for the improvement in the SR-Ca^2+^ load and the resulting better systolic Ca^2+^ release in *Nod1*^-/-^-PMI cells under isoproterenol administration.

### mRNA Levels of the β1 and β2 Adrenergic Receptors Are Elevated in Nod1^-/-^-PMI Hearts

**FIGURE 4:**
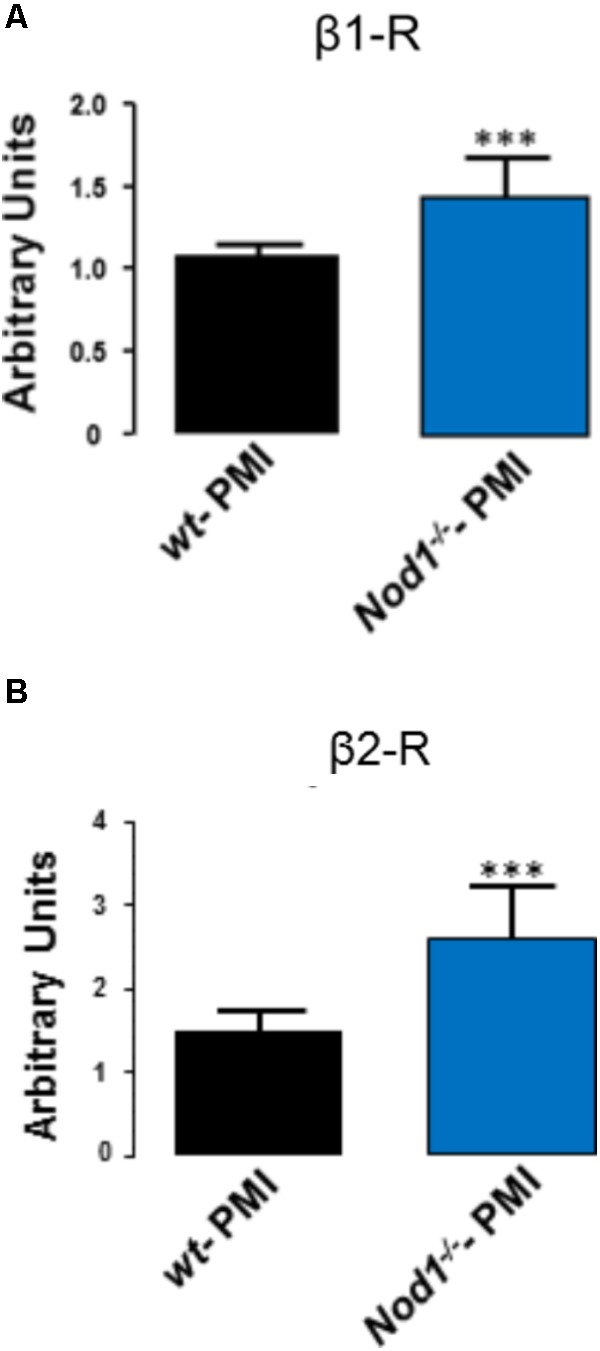
Hearts from *Nod1*^-/-^-PMI mice have elevated mRNA levels of β1 and β2 adrenergic receptors. Histograms illustrate mRNA levels of β1 **(A)** and β2 **(B)** adrenergic receptors normalized to 18s RNA levels obtained in WT-PMI (*N* = 6) and *Nod1*^-/-^-PMI hearts (*N* = 6). Values are expressed in arbitrary units. ^∗∗∗^*P* < 0.001 vs WT-PMI.

Analysis of the mRNA levels of β1 and β2 adrenergic receptors in hearts from WT-PMI and *Nod1*^-/-^-PMI mice revealed significantly increased expression of both receptors in *Nod1*^-/-^-PMI hearts (**Figures 4A,B**). These increased β1 and β2 adrenergic receptor levels can explain at least in part the improvement in Ca^2+^ regulation observed in the *Nod1*^-/-^-PMI group under isoproterenol administration.

### Selective Activation of NOD1 Compromises the β-Adrenergic Regulation of Ca^2+^ Handling in Wild-Type Cardiomyocytes

**FIGURE 5:**
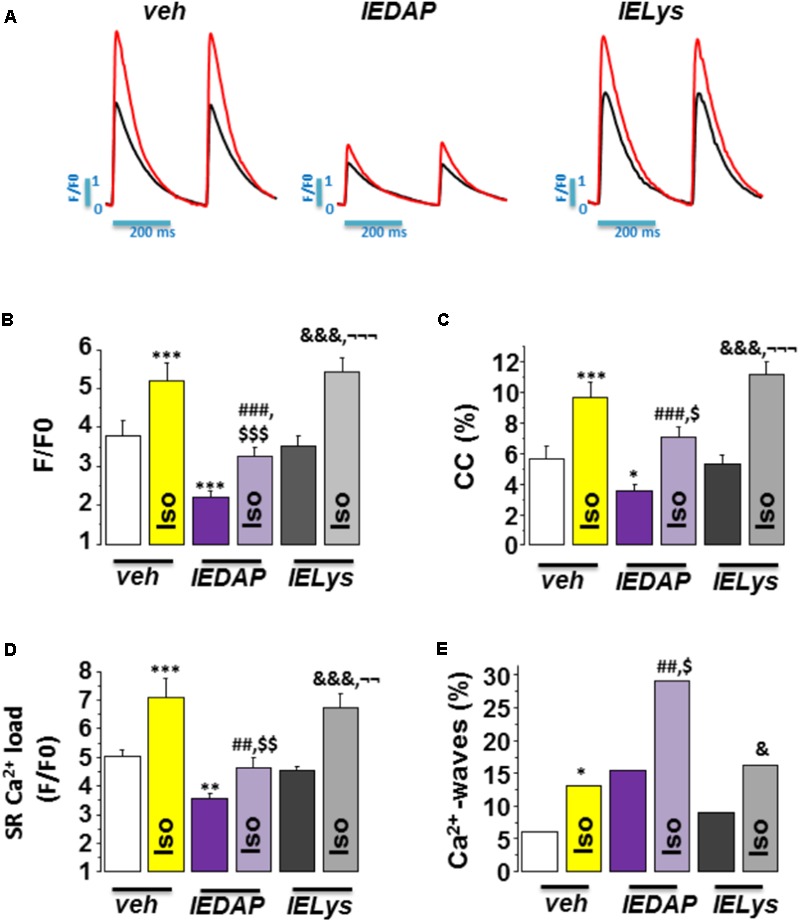
Effect of Isoproterenol on [Ca^2+^]_i_ transients, SR-Ca^2+^ load, and diastolic Ca^2+^ release in wild-type cardiomyocytes treated with C12-iE-DAP. **(A)** Representative [Ca^2+^]_i_ transients recorded in cardiomyocytes treated with vehicle (left), C12-iEDAP (20 μg/mL IEDAP, middle), and iE-LYS (20 μg/mL IELYS, right), with (red) or without (black) Iso. Mean values of **(B)** peak fluorescence [Ca^2+^]_i_ transients, **(C)** cell contraction, **(D)** caffeine-evoked [Ca^2+^]_i_ transients amplitude (SR-Ca^2+^ load), and **(E)** Ca^2+^ wave occurrence in cardiomyocytes treated with vehicle (veh; *n* = 15 cells/7 mice), C12-iE-DAP (IEDAP *n* = 16 cells/6 mice), or with iE-Lys (IELYS, *n* = 14 cells/5 mice) in the absence or the presence of Iso. Histograms represent the mean ± SEM: ^∗^*P* < 0.05, ^∗∗^*P* < 0.01, ^∗∗∗^*P* < 0.001 vs veh; ^##^*P* < 0.01, ^###^*P* < 0.001 vs IEDAP; ^&^*P* < 0.05, ^&&&^*P* < 0.001 vs IELYS; ^$^*P* < 0.05, ^$$^*P* < 0.01, ^$$$^*P* < 0.001 vs veh plus Iso; and ^¬¬^*P* < 0.01, ^¬¬¬^*P* < 0.001 vs IEDAP plus Iso.

We next addressed whether the selective activation of NOD1 impairs the β-adrenergic regulation of Ca^2+^ handling in healthy cardiomyocytes. To do this, we pretreated WT cardiomyocytes for 1 h with the NOD1 ligand C12-iE-DAP (iEDAP; 20 μg/mL) and then we examined intracellular Ca^2+^ dynamics in the absence or presence of isoproterenol perfusion. Under isoproterenol stimulation, iE-DAP-treated cells showed reduced amplitude of [Ca^2+^]_i_ transients (**Figures 5A,B**), cell contraction (**Figure 5C**), and SR-Ca^2+^ load (**Figure 5D**) compared with vehicle-treated cardiomyocytes. Treatment of cells with the inactive analog of IE-DAP, iE-Lys (20 μg/mL), resulted in a similar isoproterenol response for Ca^2+^ handling to vehicle-treated cells, demonstrating the selective effect of iE-DAP on Ca^2+^ handling under β-adrenergic stimulation (**Figures 5A–D**).

Finally, we investigated whether the selective activation of NOD1 impairs diastolic Ca^2+^ release under β-adrenergic stimulation. As shown in **Figure 5E**, isoproterenol perfusion promoted a significant increase in Ca^2+^ waves in WT cells pretreated with iE-DAP as compared with vehicle- or iE-Lys-treated cells. Importantly, the percentage of Ca^2+^ waves in cells that were pretreated with iE-DAP and under isoproterenol perfusion was very similar to that found in WT-PMI cardiomyocytes under the same conditions (34.07% in WT-PMI cells and 29.20% in cells treated with iE-DAP; compare **Figures 3C, 5E**). Supporting these data, iE-DAP administration failed to increase the percentage of Ca^2+^-wave-positive Nod1^-/-^ cardiomyocytes (data not shown).

Overall, these results indicate that NOD1 activation limits the β-adrenergic regulation of Ca^2+^ handling due to impairment of systolic Ca^2+^ release ([Ca^2+^]_i_ transients) and SR-Ca^2+^ load, which are highly conditioned by the increased diastolic Ca^2+^ leak.

## Discussion

Over the last decade, a large number of studies have shown that compensatory upregulation of the adrenergic nervous system response is a key factor in disease progression in chronic failing hearts. During the early stages of HF, there is a steady activation of the sympathetic nervous system to maintain cardiac output (Lymperopoulos, 2013), whereas in later stages, continuous adrenergic stimulation triggers negative feedback regulation of β-receptor activity, causing a downregulation in the number of receptors and also functional impairment, a process termed receptor desensitization (Böhm et al., 1990; Madamanchi, 2007), ultimately contributing to toxicity and worsening of cardiac outcomes.

Some innate immune mediators, such as NLRs, play an important role in the host response to cardiac damage. Among them, NOD1 is expressed and functional in the heart (Fernández-Velasco et al., 2012; Delgado et al., 2015) and is upregulated in failing hearts both in a mouse HF model and in failing human myocardium (Val-Blasco et al., 2017a). Genetic deletion of NOD1 prevents HF-related cardiac dysfunction through modulation of EC coupling, chiefly by improving systolic Ca^2+^ release and by maintaining Ca^2+^ SR load, which are both compromised in HF (Val-Blasco et al., 2017a).

Our present results show that deficiency of NOD1 does not affect the regulation of Ca^2+^ handling by β-adrenergic stimulation under physiological conditions, as systolic Ca^2+^ release and SR-Ca^2+^ load were similar between WT and *Nod1*^-/-^ cardiomyocytes. In the failing heart, however, deficiency of NOD1 prevents the depression of [Ca^2+^]_i_ transients and cell contractility due to maintained SR Ca^2+^ load. Accordingly, during β-adrenergic stimulation, deficiency of NOD1 leads to a significant improvement in systolic Ca^2+^ release, cell contraction parameters, and SR Ca^2+^ load in PMI cardiomyocytes, with levels mirroring those found in isoproterenol-stimulated WT and healthy *Nod1*^-/-^cells. These results are interesting given that the decreased β-adrenergic function in HF plays a key role in the compromised cardiac contraction.

Several groups have shown that other pro-inflammatory mediators including TNFα, CXR4 or IL-1β impair the β-adrenergic response and reduce cardiac contractility (Gulick et al., 1989; Finkel et al., 1992; Schulz et al., 1995; Murray and Freeman, 1996; Pyo et al., 2006), and short-term treatment of rat cardiomyocytes with the pro-inflammatory cytokines IL-1α and TNFα blocks isoproterenol-induced increases in cell contractility (Bick et al., 1997). These findings are in agreement with an early report implicating IL-1β in uncoupling β-adrenergic responses in cardiomyocytes (Gulick et al., 1989). Consistent with these data, our study shows that a selective NOD1 agonist induces a minor but significant effect on cell contractility in isoproterenol-treated cardiomyocytes. NOD1 activation also reduced the β-adrenergic modulation of systolic Ca^2+^ release due to reduced SR-Ca^2+^ load, and by increasing the diastolic Ca^2+^ leak. These results can be explained by the fact that NOD1 activation in cardiomyocytes induces the over-phosphorylation of RyR2, promoting the open state of the receptor and inducing Ca^2+^ release from the SR to the cytoplasm, increasing the diastolic Ca^2+^ leak (waves), and compromising the SR-Ca^2+^ load (Delgado et al., 2015; Val-Blasco et al., 2017a).

In many cases, increased diastolic Ca^2+^ release is the cause of depressed SR-Ca^2+^ load in HF. Our data show that during β-adrenergic stimulation, diastolic Ca^2+^ release is significantly increased in failing WT cells, whereas deficiency of NOD1 prevents the increase in the HF-related diastolic Ca^2+^ waves. These data go some way to explain the improvement in the SR-Ca^2+^ load and the resulting improved systolic Ca^2+^ release and cell contractility in *Nod1*^-/-^-PMI cells under isoproterenol stimulation.

*Nod1*^-/-^-PMI hearts showed physiological levels of phospho-RyR2 (Val-Blasco et al., 2017a), thus contributing to block Ca^2+^ diastolic leak in *Nod1*^-/-^-PMI cardiomyocytes. Aberrant diastolic Ca^2+^ leak in many cases such as HF leads to arrhythmogenesis and cardiac dysfunction (Reiken et al., 2003; Ai et al., 2005; van Oort et al., 2010; Marks, 2013; Terentyev et al., 2014). RyR-derived Ca^2+^ leak can cause arrhythmias by Ca^2+^ waves that trigger delays after depolarizations (Lehnart et al., 2006). Pro-arrhythmogenic events are common in failing patients, particularly under β-adrenergic stimulation, and around 30–50% of these individuals die from sudden cardiac death, mostly associated with ventricular arrhythmias. Our data suggest that blocking NOD1 can be a new tool to prevent ventricular arrhythmias and cardiac dysfunction resulting from Ca^2+^ mishandling in HF.

In line with our results, elevated levels of the inflammatory cytokines TNFα and IL-1β have been reported in both tissue and plasma from HF patients with arrhythmias (Levine et al., 1990; Testa et al., 1996; Aukrust et al., 2004). Moreover, other inflammatory mediators such as Toll-like receptors (TLRs) have been implicated in the generation of ventricular and atrial arrhythmias (Katoh et al., 2014; Monnerat-Cahli et al., 2014; Gurses et al., 2016). Importantly, TLRs seem to play a role in the regulation of the β-adrenergic response. Accordingly, Kizaki et al. (2008) demonstrated that TLR4 stimulation induces a down-regulation of β2 receptors in macrophages (Wang et al., 2009). In this regard, our results demonstrate that hearts from *Nod1*^-/-^-PMI mice have higher mRNA levels of β1 and β2 adrenergic receptors than WT-PMI hearts, suggesting a possible mechanism for the larger systolic Ca^2+^ release and SR Ca^2+^ reuptake in by *Nod1*^-/-^-PMI mice. β-adrenergic receptors are activated through heterotrimeric G proteins and subsequent activation of the adenylyl cyclase, which modulates the activity of several proteins essential for EC Ca^2+^ coupling, such as I_CaL_, RyR_2_, and phospholamban (Mayourian et al., 2018). Therefore, the elevated β-adrenergic receptor levels in *Nod1*^-/-^-PMI cells may increase the efficiency of cardiac EC coupling through the augmented function of I_CaL_, RyR, and SERCA, improving both systolic Ca^2+^ release and SR Ca^2+^ loading in failing hearts.

One of the most common treatments to manage β-adrenergic impairment in HF patients is the use of β-blockers. It has been demonstrated that β-blocker treatment reduces the detrimental effects of catecholamine stimulation, such as toxicity, pathological elevated heart rate, and adverse remodeling and arrhythmia development in failing patients. Improving β-adrenergic regulation of Ca^2+^ handling by blocking NOD1 can be a new tool to improve the cardiac function in failing hearts.

## Conclusion

Our study demonstrates that deficiency of NOD1 improves the β-adrenergic modulation of Ca^2+^ handling in isolated cells from failing mice. Therefore, NOD1 emerges as a new potential target in the treatment of cardiac dysfunction and ventricular arrhythmias associated with HF.

## Author Contributions

MF-V conceived, designed, and discussed the experiments and wrote the paper. AV-B, JN-G, MT, PP, CZ, MP, LR-R, and MG-F performed the experiments. MF-V, AV-B, JN-G, CD, GR-H, and PP analyzed the data. MF-V, LB, GR-H, and CD contributed reagents, materials, and analysis tools.

## Conflict of Interest Statement

The authors declare that the research was conducted in the absence of any commercial or financial relationships that could be construed as a potential conflict of interest.
