# Supplementary material for: Deficiency of NOD1 Improves the β-Adrenergic Modulation of Ca2+ Handling in a Mouse Model of Heart Failure
Source: Front Physiol. 2018 Jun 14;9:702. doi: 10.3389/fphys.2018.00702 (PMC6010671; doi:10.3389/fphys.2018.00702)
Supplement: Supplementary file 1 [file Image_1.pdf]

Supplementary Figure S1

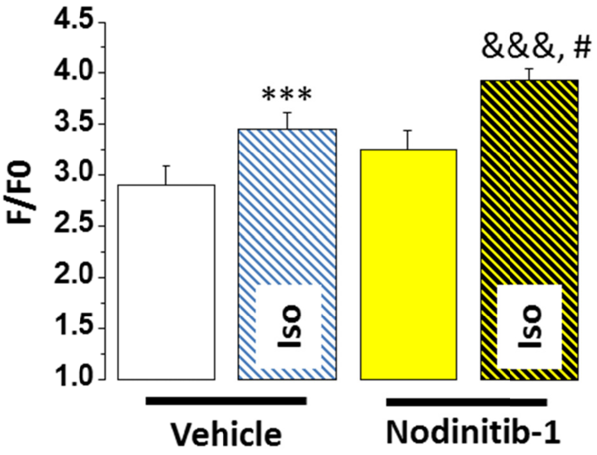

Supplementary Figure S1. Pharmacological blockade of NOD1 improves the isoproterenol modulation of systolic Ca^{2+} release in WT-PMI mice. Histograms represent mean values of peak fluorescence $[Ca^{2+}]_i$ transients (F/F0) obtained in cardiomyocytes isolated from WT-PMI mice were treated for 6 weeks (3 times weekly) with the NOD1 inhibitor**,** Nodinitib-1 (5 µmol/L; n=9/N=3) or vehicle (n=8/N=3); before and after Isoproterenol (Iso) administration (3-5 min). ****P* < 0.001 vs WT-PMI treated with Vehicle; $^{\&\&\&}P < 0.001$ vs WT-PMI treated with Nodinitib-1; $^{\#}P < 0.05$ vs WT-PMI treated with Vehicle and perfused with Iso.
